# Functional and Clinical Proteomic Exploration of Pancreatic Cancer

**DOI:** 10.1016/j.mcpro.2023.100575

**Published:** 2023-05-19

**Authors:** Peiwu Huang, Weina Gao, Changying Fu, Ruijun Tian

**Affiliations:** Department of Chemistry and Research Center for Chemical Biology and Omics Analysis, School of Science, Southern University of Science and Technology, Shenzhen, China

**Keywords:** pancreatic cancer, functional proteomics, clinical proteomics, spatial proteomics, tumor microenvironment, cancer-stroma crosstalk, post-translational modifications, exosome

## Abstract

Pancreatic cancer, in most cases being pancreatic ductal adenocarcinoma (PDAC), is one of the most lethal cancers with a median survival time of less than 6 months. Therapeutic options are very limited for patients with PDAC, and surgery is still the most effective treatment, making improvements in early diagnosis critical. One typical characteristic of PDAC is the desmoplastic reaction of its stroma microenvironment, which actively interacts with cancer cells to orchestrate key components in tumorigenesis, metastasis, and chemoresistance. A global exploration of cancer-stroma crosstalk is essential to decipher PDAC biology and design intervention strategies. Over the past decade, the dramatic improvement in proteomics technologies has enabled the profiling of proteins, post-translational modifications (PTMs), and their protein complexes at unprecedented sensitivity and dimensionality. Here, starting with our current understanding of PDAC characteristics, including precursor lesions, progression models, tumor microenvironment, and therapeutic advancements, we describe how proteomics contributes to the functional and clinical exploration of PDAC, providing insights into PDAC carcinogenesis, progression, and chemoresistance. We summarize recent achievements enabled by proteomics to systematically investigate PTMs-mediated intracellular signaling in PDAC, cancer-stroma interactions, and potential therapeutic targets revealed by these functional studies. We also highlight proteomic profiling of clinical tissue and plasma samples to discover and verify useful biomarkers that can aid early detection and molecular classification of patients. In addition, we introduce spatial proteomic technology and its applications in PDAC for deconvolving tumor heterogeneity. Finally, we discuss future prospects of applying new proteomic technologies in comprehensively understanding PDAC heterogeneity and intercellular signaling networks. Importantly, we expect advances in clinical functional proteomics for exploring mechanisms of cancer biology directly by high-sensitivity functional proteomic approaches starting from clinical samples.

## Highlights

• Post-translational modifications–mediated intracellular signaling pathways.
• Secreted ligands and plasma membrane receptors-mediated intercellular signaling.
• Tissue and plasma proteomics for the discovery of biomarkers and therapeutic targets.
• Spatial proteomics for understanding tumor heterogeneity.

## Graphical Abstract

**Figure:**
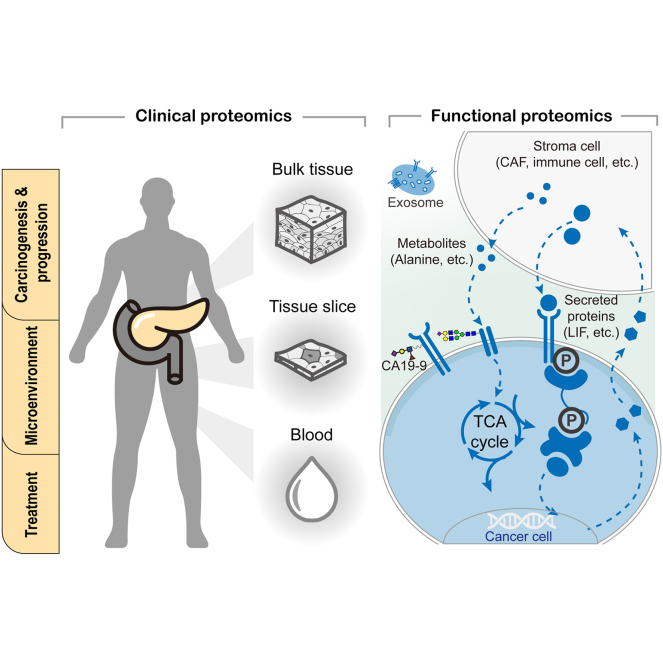

## In Brief

Pancreatic cancer is extremely lethal with limited therapeutic options. Identifying effective early-detection biomarkers and novel therapeutic targets is urgently needed. In this review, we describe recent advances in functional and clinical proteomic analyses of pancreatic cancer. Regarding functional proteomics, we highlight post-translational modifications–mediated intracellular signaling, and ligands and plasma membrane receptors–mediated intercellular signaling. In terms of clinical proteomics, we highlight bulk tissue and plasma proteomics for identification of diagnostic and therapeutic targets and spatial proteomics for studying tumor heterogeneity.

Pancreatic ductal adenocarcinoma (PDAC), which comprises a majority of pancreatic cancers, is one of the most lethal diseases with a median survival time of less than 6 months (1). With limited therapeutic options, PDAC is likely to become the second leading cause of cancer-related mortality by 2040 (2). As specific symptoms are rarely exhibited and clinically used biomarkers (*e.g.*, CA19–9) lack enough sensitivity and specificity for early detection, approximately 80% of tumors are detected at metastatic stages and are not surgically resectable; for patients who receive surgical resection and subsequent adjuvant therapy, a majority will relapse (3). Current cytotoxic chemotherapies, such as FOLFIRINOX and gemcitabine, only extend patient survival in the range of months, and the development of targeted therapies for PDAC is extremely challenging due to complicated tumor heterogeneities (4, 5, 6). A prominent pathological characteristic of PDAC is the typical desmoplastic reaction of the tumor microenvironment (TME), which is due to the deposition of extracellular matrix (ECM) by the highly abundant cancer-associated fibroblasts (CAFs) in the TME, which occupy the majority of the tumor mass (7). This highly fibrotic TME and multilayered interplay with cancer cells pose a barrier for preventing the infiltration of drugs (8, 9). Due to the heterogeneity of different stroma subpopulations, deconstructing the stroma contents has been shown to have controversial effects on either inhibiting or promoting tumor progression (10, 11). Thus, systematically exploring the heterogeneity of stromal cells and their functional crosstalk with cancer cells is critical for improving the understanding of their tumor-supportive and tumor-suppressive capacity, and facilitating the development of early diagnostic biomarkers and effective targeted therapies.

Extensive efforts have been made to study the genomic and transcriptomic landscapes of PDAC and provide insight into mutational mechanisms, molecular subtypes, and potential therapeutic targets for precise treatments (12, 13). Characterizing genetic changes alone is insufficient to interpret disease occurrence and development, including in PDAC. Proteins are the direct building blocks of biological processes, and they are major drug targets. Mass spectrometry (MS)-based proteomics is essential to bridge the gap between PDAC phenotype and genotype. Over the past years, dramatically improved proteomic technologies and analytical platforms have enabled global profiling of proteins, post-translational modifications (PTMs), and their protein complexes at unprecedented sensitivity and dimensionality (14, 15). Here, we first provide a brief introduction of our current understanding of PDAC characteristics, including precursor lesions, progression models, the tumor microenvironment, and therapeutic advancements. Then, we describe how proteomics contributes to the functional and clinical exploration of PDAC, providing insights into PDAC carcinogenesis, progression, and chemoresistance. We summarize recent achievements enabled by proteomics to systematically investigate PTMs-mediated intracellular signaling in PDAC, cancer-stroma interactions, and potential therapeutic targets revealed by these functional studies. We also highlight proteomic profiling of clinical tissue and plasma samples to discover and verify useful biomarkers that can aid in the early detection and molecular classification of patients. In addition, we introduce spatial proteomic technology and its applications in PDAC for deconvolving tumor heterogeneity. Finally, we discuss future prospects of applying new proteomic technologies in comprehensively understanding PDAC heterogeneity and intercellular signaling networks. Importantly, we expect advances in clinical functional proteomics for exploring cancer biology directly by high-sensitivity functional proteomic approaches starting from clinical samples.

## The Characteristics of PDAC

### PDAC Carcinogenesis and Progression

Compared to patients with other types of cancer, patients with PDAC often have a dismal prognosis, which is a consequence of their late diagnosis. Quantitative analysis of the genetic evolution trajectory of pancreatic cancer metastases shows that (1): it takes at least 10 years from the occurrence of initial mutation to the birth of the originator cell (2); it may take at least five more years for metastasis initiation; and (3) there may be approximately 2 years between metastasis and death (16). In most individuals, the progression of initiation alterations in the ductal epithelium is entirely somatic, and only about 10% of patients with PDAC meanwhile have the reproductive system tendency of malignant tumors (17). Genetic alterations in primary PDAC tumors include somatic mutations in driver genes, such as point mutations, which often occur in four main driving genes, including KRAS in about 90% of PDAC, TP53 in 80%, CDKN2A in 60% and SMAD4 in 40% (17). In the progression of PDAC, KRAS mutations occur in some normal ductal epithelial cells and almost all low-grade pancreatic intraepithelial neoplasias (PanINs), and high-grade PanINs are more likely to contain changes in tumor suppressor genes, such as CDKN2A, while the highest-grade lesions are accompanied by TP53 and SMAD4 loss (17, 18). However, somatic mutation alone cannot fully explain the biological and clinical differences in PDAC tumors. Some histologically normal ductal epithelium and PanIN often share similar somatic mutations with PDAC, while KRAS, the most frequent somatic mutation, has also been found in patients with chronic pancreatitis. In fact, patients with chronic pancreatitis are at an increased risk of PDAC compared with those not. Due to their similarities in clinical, radiological, and biochemical nature, misclassification of PDAC or PanIN as chronic pancreatitis has also led to a dismal prognosis. Therefore, it is necessary to systematically explore many other factors leading to PDAC heterogeneity.

### PDAC TME

Cancer hijacks nontransformed tissue stroma to create a favorable TME for tumor carcinogenesis and progression. Although immunotherapeutic strategies demonstrating ground-breaking treatment for multiple cancer types in the past decade are the popular option in PDAC clinical trials, they have limited responses in patients with PDAC. In fact, PDAC is characterized by a dynamic evolution of the most representative TME during tumor progression, which consists of abundant CAFs, immune cells, endothelial cells, compressed vessels, neurons, and ECM components (19). Depletion of stromal tissue by inhibiting the Hedgehog cellular signaling pathway increased the delivery efficiency of gemcitabine into tumors, leading to the stabilization of the disease (10). Inhibitor-targeting focal adhesion kinases (FAKs), a principal driver of the desmoplastic reaction, has also been shown to reduce tumor fibrosis, progression, and metastasis and improve the survival of a genetically engineered PDAC mouse model (20). However, others have shown that deconstructing the surrounding desmoplastic stroma could also accelerate PDAC progression in mouse models and patients (11, 21, 22). These studies reveal the heterogeneity of stromal contents (*e.g.*, different subtypes of CAFs), which have diverse functions and increase the complexity and difficulty of directly targeting the stroma for PDAC therapy.

The role of CAFs in promoting or inhibiting tumor occurrence and progression is affected by the surrounding intercellular signals (9). Nonepithelial cells in the TME communicate with each other to create a unique network and produce a large amount of ECM components, including growth factors, matricellular proteins, catalytically active enzymes, and cytokines (19). The interaction between tumor cells and stroma is not unidirectional but rather bidirectional or even multidirectional because stromal cells have been proven to significantly affect cancer cell survival by promoting proliferation and inhibiting apoptosis, while cancer cells also support stroma growth through the transformation of normal fibroblasts (23, 24). It is therefore important to establish a systematic understanding of the intercellular crosstalk between cancer cells and stromal cells mediated by dynamic pools of signaling machinery and determine how the machinery spatiotemporally controls downstream intracellular processes (25).

### PDAC Treatment

**Fig. 1:**
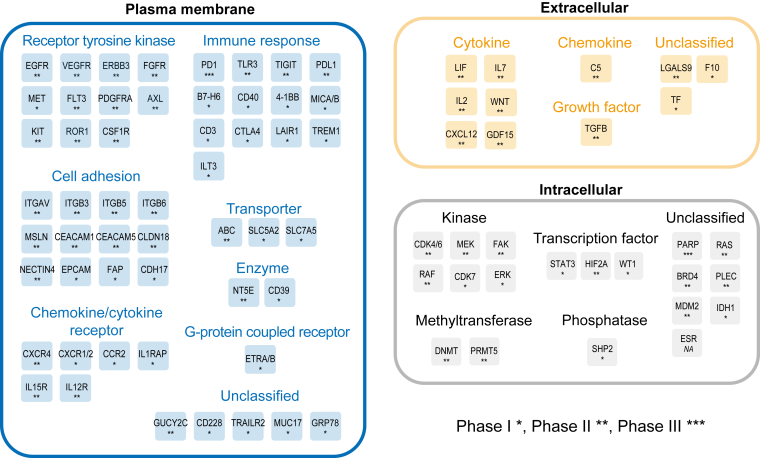
**Protein targets of clinical trials in the last 3 years that specifically investigated PDAC or included patients with PDAC.** Proteins are classified according to their subcellular locations and molecular functions. The phases of clinical trials are indicated by the number of *asterisk(s)*.

Currently, cytotoxic chemotherapy is still the main treatment for PDAC (26). For example, FOLFIRINOX, a regimen consisting of leucovorin calcium, fluorouracil, irinotecan hydrochloride, and oxaliplatin, is the standard-of-care adjuvant therapy approved by FDA for patients with resectable PDAC. The combination of gemcitabine with capecitabine or nab-paclitaxel has also been demonstrated in clinical trials to significantly improve overall survival for patients with resected and metastatic PDAC compared to gemcitabine monotherapy (27, 28). For patients at advanced or metastatic stages, somatic mutations and chromosomal rearrangements generally occur. Some drugs have received FDA approval for treating patients with genetic aberrations, such as pembrolizumab and larotrectinib, which target mismatch repair deficiencies (4). However, these genetic alterations are rare, thus continuous development of targeted therapies is highly desirable. With respect to therapies targeting aberrantly activated proteins in tumors, we summarized the protein targets of clinical trials in the last 3 years that specifically investigated PDAC or included patients with PDAC (Fig. 1 and supplemental Table S1). A majority of clinical trials target proteins localized at the plasma membrane, such as receptor tyrosine kinases (RTKs), cell adhesion proteins, and immune response-related proteins, mainly due to their important roles in cancer signaling and easy accessibility by drugs. The extracellular proteins targeted by clinical trials are mainly secreted ligands, such as cytokines, chemokines, and growth factors. These proteins are key drivers of cell-autonomous and intercellular signaling, the inhibition of which likely blocks cancer-stroma crosstalk. Kinases are the main type of intracellular proteins targeted by clinical trials, such as CDKs, which are crucial for controlling the cell cycle during cell differentiation and proliferation, and FAKs, which are closely related to ECM production. Other proteins, such as PARP, which plays a key role in DNA damage repair, are also hot targets for clinical trials.

## Functional Proteomics of PDAC

### Exploration of PTMs-Mediated Signaling Pathways in Cancer Cells

Dysregulated PTMs can lead to abnormal protein function, localization, turnover, and many other activities. The identification of altered PTMs is critical for revealing the molecular mechanisms and identifying effective therapeutic targets of PDAC. Among a wide range of more than 300 PTMs, the most studied PTMs in PDAC include but are not limited to phosphorylation and glycosylation.

**Fig. 2:**
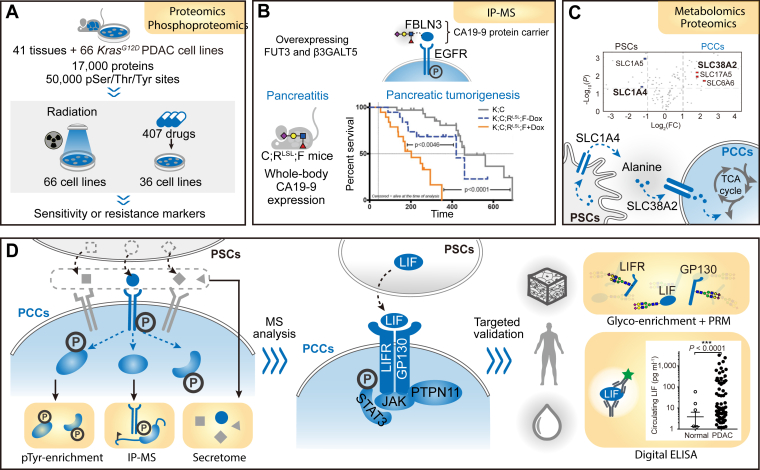
**Representative functional proteomic studies of PDAC.***A*, correlation analysis of global proteomic and phosphoproteomic datasets of 41 healthy tissues and 66 mouse Kras^G12D^ cell lines with their phenotypic data in response to radiation and drugs. *B*, IP-MS enabled identification of CA19-9 protein carriers, including FBLN3, modification of which by CA19-9 leads to EGFR hyperactivation and induction of pancreatitis and PDAC carcinogenesis. *C*, combination of metabolomics and proteomics revealed critical roles of transporters in alanine-mediated cancer-stroma crosstalk. *D*, generic and combinatory functional proteomics revealed LIF as a critical cytokine in stromal activation of cancer cell signaling, and targeted proteomics and digital ELISA validated LIF as a plasma biomarker for PDAC detection. Adapted with permission from References (31) (*A*), (34) (*B*), (41) (*C*), and (43) (*D*), respectively.

Phosphorylation is a reversible PTM that is crucial to the regulation of inter- and intracellular signaling networks. A recent study performed global phosphoproteomic analysis to identify KRAS-driven signaling pathways in human PDAC cell lines and revealed hyperactivation of cyclin-dependent kinases (CDKs) as important regulators of KRAS dependency. Drug library screening of 294 FDA-approved or clinical trial-tested compounds in KRAS-independent and -dependent cancer cell lines identified AT7519, a CDK 1, 2, 7, and 9 inhibitor as a potent inhibitor of the viability of KRAS-dependent cells. Moreover, AT7519 suppressed the growth of patient-derived xenografts (PDXs) and organoids carrying KRAS mutation by blocking phosphorylation of CDK substrates and inducing apoptosis (29). Another study generated global proteomic and phosphoproteomic datasets of tissues from PDAC mouse models to identify Kras^G12D^-driven signaling networks (30). Bioinformatics analysis revealed the effect of KRAS activation on signaling consequences, and statistically humanized pathways by comparison with public genomic and proteomic data of human PDAC. A quantitative proteome and phosphoproteome draft of the mouse was recently constructed, covering 17,000 genes and 50,000 phosphorylation sites across 41 healthy tissues and 66 Kras^G12D^ mouse PDAC cell lines (31). To leverage the mouse proteome data, the authors performed statistical analysis to link the proteome and phosphoproteome data with the phenotypic data of the cell lines responding to radiation (five doses, 66 cell lines) and drugs (407 drugs, seven doses, 36 cell lines), and established reliable models based on identified candidate markers to predict responses to radiation and drugs (Fig. 2*A*). Humphrey and coworkers profiled phosphotyrosine (pTyr) of two large panels of human pancreatic cancer cell lines, including 19 lines from ATCC and 17 primary PDX lines from The Kinghorn Cancer Centre (TKCC), and quantified over 1800 class 1 pTyr sites (32). Hierarchical clustering classified the ATCC cell line series into subtypes with enriched signaling networks related to epithelial-to-mesenchymal transition (EMT), mRNA metabolism, and RTK signaling, while the TKCC series was clustered into “low pTyr”, “mixed”, and “RTK enriched” subtypes.

Glycosylation is another common PTM, especially for membrane receptors and secreted ligands. Apart from modulating protein structure and stability, glycosylation has been implicated in regulating signaling pathways and is closely related to carcinogenesis and tumor progression (33). The only FDA-approved PDAC biomarker, carbohydrate antigen 19-9 (CA19-9), is a glycan. To investigate the role of CA19-9 in contributing to pancreatic disease pathogenesis, Engle *et al*. overexpressed fucosyltransferase 3 (FUT3) and β1,3-galactosyltransferase 5 (B3GALT5) in mouse PDAC cells to enable CA19-9 production (34). Encouragingly, IP-MS identified many CA19-9 protein carriers, 72.3% of which overlapped with those identified in human PDAC cell lines, such as MUC1, MUC5AC, CD44, and FBLN3. Upregulation of CA19-9 in transgenic mice expressing FUT3 and B3GALT5 rapidly induced severe pancreatitis, which was reversed by CA19-9 antibodies. Functionally, CA19-9 regulated EGFR hyperactivation by modifying FBLN3 to increase its binding with EGFR, leading to the induction of pancreatitis. In addition, CA19-9 elevation-induced pancreatitis accelerated pancreatic tumorigenesis by cooperating with the Kras^G12D^ oncogene, highlighting CA19-9 as a therapeutic target in PDAC (Fig. 2*B*). In a comprehensive proteogenomic study of PDAC, TMT-based proteomic, phosphoproteomic, and intact glycoproteomic analyses were performed on tumor tissues and adjacent nontumor tissues collected from 140 patients with PDAC (35). For glycoproteomic analysis, more than 34,000 intact glycopeptides (approximately 1500 N-glycosites per sample) were identified. The levels of certain glycan modifications were positively correlated with the expression levels of corresponding glycosylation enzymes. For example, glycosylation of fucosylated and/or sialylated glycans on proteins upregulated in tumors was correlated with upregulation of FUT3/FUT11 and ST6GAL1/ST3GAL1, enzymes responsible for fucosylation and sialylation, respectively. The results indicate that inhibiting these enzymes might be therapeutically beneficial, as these two glycan branches were most common on tumor-upregulated glycoproteins. For phosphoproteomic analysis, 51,469 phosphosites were identified and applied to stratify five kinases (AKT1, CDK7, PAK2, PAK1, and SRC) downstream of activated KRAS as potential therapeutic targets with known inhibitors already approved by FDA or under investigation.

In summary, MS-based proteomic characterization of phosphorylation and glycosylation in PDAC cell lines and tissues has advanced our understanding of PDAC biology and pathology, revealed functional proteins as therapeutic targets, and provided rich resources for validating other vulnerabilities. In addition to phosphorylation and glycosylation, several common PTMs, such as ubiquitination and acetylation, are also altered in PDAC and play critical roles in tumorigenesis, progression, and metastasis. Integrated analysis of different PTMs and the crosstalk between them will enable deep biological and clinical insights and has the potential to identify combinatorial therapeutic targets.

### Exploration of Cancer Cell Signaling Mediated by Membrane Proteins

In multicellular organisms, plasma membrane (PM) proteins are responsible for the initiation of cell-autonomous signaling pathways and cell–cell interactions. The binding of ligands to PM receptors leads to the activation of intracellular signaling and the manifestation of corresponding cell phenotypes. In addition, PM proteins are an important source for the discovery of biomarkers and therapeutic targets, as they are favorable for exposure to antibody drugs (36). In PDAC, oncogenic KRAS physically and functionally interacts with PM proteins. To systematically explore the PDAC surfaceome regulated by KRAS signaling, cell surface proteins of KRAS-ON and KRAS-OFF cells were biotinylated with a sulfo-NHS-SS-biotin probe and chromatographically isolated for surfaceome comparison by SILAC-based proteomic analysis (37). Among 221 differentially expressed PM proteins, SDC1 was validated as a key regulator of macropinocytosis-mediated metabolic reprogramming driven by the KRAS-MAPK-PSD4-ARF6 signaling axis. EGFR upregulation frequently occurs in various types of tumors and is associated with poorer prognosis. Choi *et al*. found that EGFR activation in pancreatic cancer cells (PCCs) was mediated by galectin-3 binding protein (Gal-3BP), a secreted protein identified by TMT-based quantitative proteomics to be highly abundant in tumor interstitial fluid (38). Co-immunoprecipitation found that Gal-3BP and galectin-3 were associated with EGFR to positively regulate its activation and mediate EMT and metastasis in PDAC. Gupta *et al*. performed affinity purification to capture lysosomes from PCCs and HEK293T cells overexpressing TMEM192, a lysosome membrane-specific protein that was fused to 3× haemagglutinin tags, for LFQ-based proteomic analysis. Myoferlin was identified as a highly abundant membrane protein of PCC-derived lysosomes (39). Functionally, Myoferlin depletion significantly suppressed tumor growth *in vivo* by damaging the lysosome membrane. Targeting Myoferlin and other membrane repair factors might therefore shed light on new therapies for PDAC.

PM transporters are gatekeepers of nutrient metabolites. Understanding the role of transporter-mediated metabolic reprogramming in promoting tumor growth and therapeutic resistance is fundamental for the discovery of therapeutic opportunities. A previous multiple reaction monitoring (MRM)-based quantitative metabolomics study found that pancreatic stellate cells (PSCs) were critical for PDAC metabolism through autophagy-mediated secretion of alanine to fuel the tricarboxylic acid (TCA) cycle and lipid biosynthesis of tumor cells (40). However, whether amino acid transporters also regulate alanine crosstalk between stromal and cancer cells is unclear. To address this, the same group first performed stable-isotope tracing-based metabolic flux analysis to reveal that PSCs mainly secreted alanine, whereas alanine uptake was more evident in PCCs (41). Then, to identify transporters involved in the differential channeling of alanine between PSCs and PCCs, TMT-labeling-based quantitative proteomic analysis was performed, and two neutral amino acid transporters, SLC1A4 and SLC38A2, were found to be highly expressed in PSCs and PCCs, respectively (Fig. 2*C*). Mechanistically, PSCs utilized SLC1A4 and other passive transporters to maintain the alanine concentration in the environment, while PCCs upregulated SLC38A2 to meet their increased demand for alanine. Inhibition of SLC38A2 suppressed tumor initiation and progression in subcutaneous and orthotopic models. To uncover the molecular mechanism of CD9-mediated promotion of tumor growth in PDAC, AP-MS was carried out to identify the CD9 interactome, and glutamine transporter ASCT2 (also named SLC1A5) was among the top hits (42). Functionally, CD9 promoted PM localization of ASCT2, leading to enhanced glutamine uptake in PCCs. Deletion of CD9 increased the response of PDAC organoids to ASCT2 inhibitors and led to prolonged survival in KPC mice, suggesting a potential therapeutic strategy targeting CD9 and glutamine metabolism.

### Stromal Activation of Cancer Cell Signaling

Diverse CAFs, lymphocytes, endothelial cells, and other cell populations comprise the stromal compartment of PDAC and contribute to both protumor and antitumor processes through diverse intercellular signaling pathways. Considering that intercellular signaling is often activated by pTyr after intercellular ligand and membrane receptor recognition, we developed a generically applicable and combinatory functional proteomic approach to systematically explore these pTyr-based intercellular signaling events and validate proteins in clinical samples (Fig. 2*D*). By collaborating with Dr Hunter’s group, we applied this approach to investigate the paracrine signaling from PSCs to PCCs (43). First, pTyr proteomic analysis of PCCs stimulated by PSC-derived conditioned medium (CM) identified STAT3 and PTPN11 to be highly activated. IP-MS analysis of the STAT3 interactome in PCCs stimulated by PSC CM revealed that LIF receptor (LIFR) and its coreceptor GP130 were the most enriched proteins. Finally, secretome analysis of PCCs and PSCs revealed LIF to be specifically secreted by PSCs, collectively suggesting that PSC-derived LIF is a key paracrine ligand that actives the PCC LIFR-GP130 complex and downstream JAK-STAT3 signaling. Functional studies in transgenic mouse models of PDAC revealed that paracrine activation of the LIF-LIFR-GP130 signaling complex was crucial in modulating EMT status and cancer cell differentiation, and blocking this signaling complex significantly reduced tumor growth and prolonged survival time. Importantly, glycoprotein enrichment combined with targeted proteomics demonstrated that this signaling complex was significantly regulated in clinical PDAC tumor tissues, and LIF was further validated as a potential blood biomarker for early detection of PDAC by developing a highly sensitive digital ELISA. Moreover, neutralizing antibody targeting LIF has entered a phase II clinical trial for PDAC in the United States, supporting the potential role of this signaling complex as new biomarkers and drug targets for PDAC.

To understand the mechanisms of the stromal effect on shaping pancreatic cancer heterogeneity, Ligorio and coworkers combined single-cell RNA sequencing (scRNA-seq), phosphoproteomics, secretome profiling, and mass cytometry to analyze PCCs co-cultured with different proportions of CAFs, and found that co-cultured CAFs drove PCCs to shift toward invasive EMT and proliferative phenotypes by secreting TGF-β1 and activating the MAPK and STAT3 signaling pathways in PCCs (44). To translate their cell line model-based findings to patients with PDAC, they performed RNA *in situ* hybridization staining of the EMT marker FN1 and proliferative marker MKI67 in a large cohort of human PDAC tumors and validated that the stromal expression of these markers was positively correlated with gland heterogeneity in PDAC tumors. Another study explored the mechanisms underlying how CAFs support PCC metabolism through FAKs (45). Proteomic and phosphoproteomic comparison of mouse PCCs treated with CM from FAK-depleted CAFs and wild-type CAFs identified significantly changed metabolic pathways, such as significantly upregulated enzymes that are critical in glycolysis. Mechanistically, FAK depletion in CAFs increased the secretion of chemokines CCL6 and CCL12, which activated protein kinase A through CCR1/CCR2, resulting in enhanced glycolysis in PCCs.

Immune cells such as macrophages (MØs) infiltrate early during PDAC development and accelerate tumor progression by suppressing antitumor immunity within the PDAC TME. To understand how MØs reprogram fibroblasts to regulate tumor growth and metastasis, Dr Jørgensen’s group performed SILAC-based quantitative phosphoproteomic analysis of PCCs treated with CM from PSC–PCC and MØ–PSC–PCC co-cultures (46). Supported by LUMINEX-ELISA and RNA-Seq analyses of PCCs, PSCs, and MØ isolated from PSC–PCC and PSC–MØ–PCC co-cultures, they found that MØ-derived Oncostatin M (OSM) activated the OSM receptor (OSMR) on PSCs and stimulated expression of inflammation-related secreted ligands, which in turn induced a protumor environment to activate proliferative and migratory signaling pathways in PCCs, implying therapeutic potential by targeting the MØs–PSC–PCC interaction through OSM–OSMR complex. In another study, secretome analysis of MØs co-cultured with apoptotic PDAC cells identified 14-3-3ζ as a crucial regulator of PDAC chemoresistance through activation of the AXL-AKT signaling pathway (47).

### Systematic Exploration of Tumor-Stroma Reciprocal Signaling

MS-based systematic exploration of the molecular regulatory mechanisms of reciprocal cancer-stroma interactions could aid in developing therapies to block tumor-promotive cancer-stroma crosstalk and improve patient outcomes. One representative proteomic study to explore reciprocal tumor-stroma signaling was conducted by Dr Jørgensen’s group (48). Using a commercial antibody array targeting 144 soluble cytokines, growth factors, and receptors, they first compared the CM between KRAS^G12D^ and KRAS^WT^ PCCs and found that KRAS^G12D^ induced secretion of the morphogen sonic hedgehog (SHH). SILAC-based quantitative proteomic analysis of PSCs stimulated by SHH revealed widespread regulation of the secretome, such as upregulation of ECM components and growth factors IGF1 and GAS6. Then, to investigate the reciprocal signaling axis back to tumor cells initiated by PSC activation, they performed TMT-based phosphoproteome comparison of KRAS^G12D^ and KRAS^WT^ PCCs treated with CM from SHH-stimulated PSCs, and the results revealed rapid activation of the IGF1R and AXL/TYRO3 signaling axes. Furthermore, they performed cell type-specific labeling with amino acid precursors (49, 50) to compare cell autonomous and reciprocal phosphoproteomes in direct PSC-PCC co-culture systems, and revealed PSC-PCC reciprocal signaling beyond autonomous pathways. In addition, the critical role of the KRAS^G12D^-driven reciprocal AXL/IGF1R-AKT signaling axis was validated in regulating tumor cell proliferation and apoptosis (48). Another recent proteomic study also identified the central role of the GAS6-AXL axis in protumor cancer-stromal cell reciprocation in PDAC (51). Collectively, recent advancements in the global understanding of the cancer-stroma interaction in PDAC have been made through MS-based proteomics. However, these studies mainly focused on only one stromal cell type, while various cell populations reside in the stroma. To obtain a comprehensive map of the stroma effect on PCC signaling and the intercellular signaling between different stromal cells, future studies will require proteomic profiling of various cell types by establishing various co-culture models and validated in relevant models to recapitulate complex cell‒cell interactions in the tissue context.

## Clinical Proteomics of PDAC

### Bulk Tissue Proteomics of PDAC

To date, most tissue proteomic data have been collected by analyzing bulk tissues. For example, to map the proteome of the human body, Jiang *et al*. performed TMT 10-Plex labeling and 2D-LC fractionation to analyze 32 normal human tissues and quantify proteins encoded by over 12,000 genes, providing a valuable resource to systematically examine tissue-specific proteins, protein localization, metabolism coordinated by multiple organs, and mechanisms of human diseases (52). In cancer research, recent improvements in MS-based proteomics enable deep and quantitative characterization of tumor tissues for clustering molecular subtypes and predicting therapeutic outcomes (53).

**Fig. 3:**
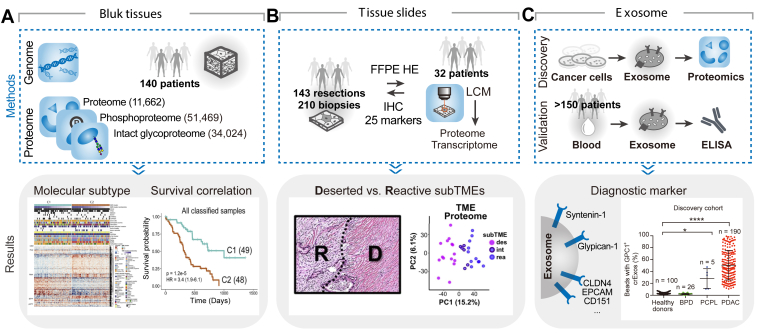
**Representative clinical proteomic studies of PDAC.***A*, Proteogenomic analysis of PDAC bulk tissues classified tumors into classical and basal-like subtypes, with the classical subtype showing a better prognosis than the basal-like subtype. *B*, LCM-based spatial proteomic and transcriptomic analyses of deserted and reactive subTMEs discovered by large-scale histological examination of PDAC slides. *C*, Proteomic analysis of cancer cell-derived exosomes identified PDAC exosome-specific proteins, which were validated as PDAC biomarkers with high specificity. Adapted with permission from References (35) (*A*), (66) (*B*), and (78, 79, 81) (*C*), respectively.

Previous molecular subtyping of PDAC was mainly carried out by genomics and transcriptomics studies (54). Recently, proteogenomic analysis of bulk tumor tissues has become a popular multi-omic analysis approach and has been successfully applied to multiple cancer types, including PDAC. As mentioned in the previous section, the most comprehensive proteogenomic study of 140 patients with PDAC was conducted by Dr Hui Zhang's group (35). Through RPLC-based fractionation into 24 fractions, a total of 11,662 proteins were identified in global proteomic analysis. In addition, sequencing of the whole genome, exome, RNAs, microRNAs, and methylation revealed the impact of genomic alterations on protein expression, PTMs, and signaling pathways. By integrating different datasets, the authors classified tumors into classical and basal-like subtypes, with the classical subtype showing a better prognosis than the basal-like subtype (Fig. 3*A*). Law *et al*. performed TMT-based proteomic profiling of 56 PDAC liver metastasis tissues and identified a total of 3960 proteins, from which the profiles of 916 proteins were used to cluster patients into four proteomic subtypes (metabolic, proliferative, progenitor-like, and inflammatory) that were associated with different clinical information, such as risk factors (smoking and drinking), survival time, and responses to drug treatment (55). A recent study performed DIA-based proteomic profiling of tumor, nontumor, PanIN, and lymph node tissues punched from different regions of FFPE tissues from 52 patients with PDAC (56). A total of 3927 proteins were identified and 2311 proteins were quantified for hierarchical cluster analysis, which separated tumor samples into three subtypes, with enriched biological processes related to adhesion, metabolic features, and splicing and nucleoplasm activity.

Bulk tissue proteomics has also been applied for the identification and verification of biomarkers for PDAC. To develop a protein features-based classification model for the stratification of patients with PDAC into risk subgroups, Son and colleagues performed stable isotope-labeled standard (SIS) peptide-based MRM-MS analysis of fresh-frozen bulk tissues collected from 244 patients with PDAC (161 samples in the training set and 83 samples in the validation set) (57). A total of 200 MRM target proteins (203 peptides) with relevance to tumor progression or prognosis were screened out from multiple sources, such as the Early Detection Research Network and the Cancer Genome Interpreter. Of the 200 proteins quantified, 24 proteins were screened stepwise according to different criteria (*e.g.*, response cure, repeatability, Pearson’s correlation) to build a random forest classification model, which classified patients into four risk subgroups (stable, exocrine-like, activated, and ECM-remodeling) associated with different patient outcomes. Sahni *et al*. performed SWATH-MS analysis of tissue samples from good-responders and poor-responders patients with PDAC under neoadjuvant chemotherapy and revealed a novel biomarker signature panel (GRP78, CADM1, PGES2, and RUXF) that could predict poor response to neoadjuvant chemotherapy (58). Kanda and colleagues performed a DIA-based proteomic analysis of tumor and adjacent normal tissues derived from patients with PDAC in low and high CA19-9 groups (59). Systematic evaluation of the dysregulated signaling pathways revealed that metabolic reprogramming was significantly different between these two groups. For validation, proteostasis related proteins EIF2 and ATF4 were confirmed by immunohistochemistry (IHC) to be significantly downregulated in tumors from CA19-9 high patients. The data thus provide cues for screening patients in clinical trials who can benefit from therapies targeting metabolism in PDAC.

Organoids are 3D cellular clusters that retain genetic and functional characteristics of the original tissues and are thus powerful alternatives for both basic and translational research on PDAC. Boj *et al*. generated organoid models from human and mouse pancreatic tissues and found that organoids orthotopically implanted into syngeneic mice could recapitulate tumor development from early-grade neoplasms to advanced carcinomas (60). Comprehensive transcriptional and iTRAQ-based quantitative proteomic analyses of murine organoids identified a total of 6051 protein groups, from which the authors revealed pathways altered during different stages of disease progression, such as glutathione metabolism and biological oxidations, in comparison of PanIN *versus* normal organoids. A recent study performed proteomic and functional protein network comparison of organoids derived from normal and neoplastic pancreas and revealed alterations in cellular programming with disease progression (61). Another recent study integrated a chromatin accessibility atlas with genomic and transcriptomic sequencing of 84 PDAC organoid lines to reveal signatures associated with drug sensitivity (62). In addition, proteome profiling of the media supernatant of organoids revealed a panel of secreted proteins (CD14, GPC4, ANXA11, and CD44v6) that were validated as circulating biomarkers for the detection of PDAC using plasma samples from patients with PDAC and benign diseases (63). These studies demonstrate that organoid models could serve as a powerful platform to not only decipher molecular mechanisms of disease progression and *in vivo* drug responses but also discover clinically actionable biomarkers. The main limitation of tissue-derived organoids for PDAC research is that only cancer cells grow massively while stromal cells are much less represented in PDAC organoids. In comparison, genetically engineered mouse KPC (Kras^LSL-G12D/+^; Trp53^flox/flox^; Pdx1-Cre) models have significant advantages over organoids. First, KPC tumors appear spontaneously and closely resemble disease stages and outline many prominent clinical features of this human disease, such as fibrosis (64). Second, KPC mice are immunoactive, providing a useful platform for studying the interaction between the immune system and tumor cells (65). Last, KPC is an ideal animal model to study PDAC development, especially at an early stage, considering that early-stage human PDAC tissues are rarely collected clinically. Thus, global and PTM proteomics of tissues from different stages of KPC models will be worthy of exploration in the future.

### Exploration of PDAC Proteome With Spatial Resolution

A key hallmark of PDAC is extensive stromal content and low tumor cellularity, especially at the advanced stage, which hampers precise capture of tumor-specific information. Therefore, cell type-resolved molecular analysis of PDAC tissues is appealing but challenging. To define precise stromal contributions to the intratumoral heterogeneity of PDAC, Grünwald *et al*. deconvoluted the PDAC TME through a large-scale histological examination of 143 resections and 210 biopsies according to differences in features of stromal cell morphology, the ratio of cellular to acellular components and the extent of ECM, and categorized deserted and reactive subTMEs that were associated with different patient outcomes (Fig. 3*B*) (66). Proteomic and transcriptomic profiling of these subTMEs harvested by laser capture microdissection (LCM) from 32 tumor tissues identified a total of 5708 protein groups and 17,945 transcripts, among which 5475 genes were detected at both protein and RNA levels. Bioinformatic analysis of differentially expressed genes revealed that deserted subTMEs were enriched with humoral immunity pathways and ECM signaling, while reactive subTMEs were enriched with signaling pathways related to cellular immunity, cellular stress response, and CAF-activation. They further validated distinct CAF differentiation states and immune phenotypes with chemoprotective and tumor-promoting functions. Le Large *et al*. reported a large-scale proteomic dataset of over 6000 proteins identified from tumor and stroma compartments harvested by LCM of 16 human PDAC tumor tissues, from which they found more pronounced protein heterogeneity in tumor areas than in the stroma (67). The expression levels of tumor-specific CALB2 and stroma-specific COL11A1 were validated by IHC analysis of tissue microarrays to be correlated with patient survival. In a study to overcome the bias due to high heterogeneity between cholangiocellular carcinoma (CCC) and PDAC tumor tissues, Padden *et al*. conducted LCM to isolate tumor cells from CCC and PDAC for proteomic analysis and validated ANXA1 and ANXA10 as biomarkers that could distinguish CCC tumors from PDAC liver metastases with high sensitivity and specificity (68). Perineural invasion (PNI) is a common characteristic of PDAC associated with the generation of pain, tumor recurrence, and poor prognosis. Proteomic analysis of PCCs and nerves harvested by LCM from five PNI and non-PNI PDAC FFPE tumors revealed that the proteomes of PNI and non-PNI PCCs were similar, while PNI nerves showed differential expression of proteins related to neuronal plasticity similar to neuronal injury (69).

The ECM comprises a bulk mass of PDAC stroma and regulates many behaviors of tumor cells. To systematically characterize changes in ECM proteins during PDAC progression, Tian *et al*. enriched ECM proteins from normal, PanIN, and tumor-bearing pancreatic tissues of both transgenic mouse models and human patients with PDAC, together with samples from CP patients, by using an optimized ECM isolation protocol (70). Tissue samples were homogenized, and ECM proteins were selectively extracted based on their insolubility, crosslinking, and large size compared with other soluble cellular proteins. The retained ECM proteins were subjected to TMT 10-plex-based quantitative proteomics analysis. Around 300 ECM proteins were identified, comprising over 90% of the sum ion intensity of precursors, while more than 2500 non-ECM proteins accounted for less than 10%, suggesting that ECM proteins were successfully enriched. The authors found that the change in ECM composition was similar between mouse models and human patients. Probing the ECM isolated from stromal and tumor cells revealed that although most of the microenvironmental ECM components were derived from stromal cells, ECM proteins secreted by cancer cells were better correlated with short patient survival time. In summary, proteomics, genomics and transcriptomics of PDAC tissues with spatial resolution have revealed subtypes of tumor and stroma compartments and stromal contributions to the intratumoral heterogeneity of PDAC. However, current spatial proteomic technologies have limitations, such as low resolution, low throughput, and low proteome coverage, and mainly focus on profiling the total proteome, which restrict their applications in large-scale, multidimensional dissection of the functional proteomic landscape of PDAC TME. Thus, continued development of the field will include high-resolution spatial proteomics, spatial characterization of protein PTMs, and multidimensional analysis of intratumoral heterogeneity and protein networks between distinct cell types.

### Plasma Proteomics of PDAC

As surgical resection remains the most effective treatment for PDAC, improvements in early detection are critical. Currently, CA19-9 is the only FDA-approved blood biomarker for the clinical management of PDAC. However, the sensitivity and specificity of CA19-9 in PDAC diagnosis are inadequate, which limits its application for general screening (71). Thus, the discovery of a biomarker or biomarker panel with increased sensitivity and specificity is urgently needed to improve the survival outcome of PDAC. Plasma is likely to contain proteins secreted from the tumor site, thus is a valuable source for the identification of tumor-specific biomarkers. However, plasma proteomic analysis remains a daunting task due to the huge dynamic range of the plasma proteome that exceeds 10 orders of magnitude. Apart from the depletion of highly abundant plasma proteins, other technologies have been introduced to address the dynamic range issue of plasma proteomics, such as engineered magnetic nanoparticles (NPs) with varying physicochemical properties, enabling reproducible formation of distinct protein corona patterns for differential adsorption and characterization of the plasma proteome (72). Improvements in MS acquisition strategies have also increased the efficiency of plasma proteome profiling. Data-independent acquisition is becoming popular for single-shot analysis to achieve deep proteome coverage and high quantification accuracy (73). Collectively, there are several types of plasma proteomics studies of PDAC according to the discovery and validation workflows.

The first type of study is aimed at discovering biomarker candidates by global plasma proteome profiling, followed by validation in much larger sample cohorts. To develop a multimarker diagnostic panel that could improve the performance of CA19-9 in clinical practice, Kim *et al*. surveyed proteomic literature and databases to screen a list of 205 proteins with area under the curve (AUC) values >0.6 as initial candidates for SIS peptide-based MRM-MS analysis (74). In the initial verification phase between 50 cases with PDAC and 84 healthy controls, 54 proteins were differentially expressed and selected for final MRM-MS analysis. Finally, they established a biomarker panel of 14 proteins with AUC = 0.977 for the training set (261 PDAC cases and 290 controls) and AUC = 0.953 for the validation set (65 PDAC cases and 72 controls), which outperformed the diagnostic performance of CA19-9 alone (AUC = 0.872 and 0.832, respectively). Liu *et al*. introduced a pipeline that combined iTRAQ-based discovery proteomics with MRM-MS to discover serum biomarkers for PDAC (75). In the discovery phase, the authors identified more than 1000 proteins from pooled serum samples after the depletion of 14 high-abundance serum proteins. In the verification and validation phase, they first optimized the MRM method using synthesized peptides of 142 differentially expressed proteins, and then performed SIS peptide-based MRM analysis of four proteins (ITIH3, APOA1, APOA1, and APOL1) in 100 serum samples (34 normal controls, 26 benign diseases, and 40 cases with PDAC). Finally, a combination of the four proteins with CA19-9 outperformed the sensitivity and specificity of CA19-9 alone (AUC = 0.99 vs. 0.78 when comparing PDAC and normal control). By adopting an Intact-Protein Analysis System for extensive protein fractionation after the depletion of the three most abundant proteins, Faca *et al*. identified 1442 plasma proteins from PDAC and PanIN mouse models, and later validated TIMP1 (AUC = 0.949) and LRG1 (AUC = 0.887) as candidates for early detection of PDAC by using serum samples from 187 PDAC cases, 93 benign pancreatic disease, and 169 healthy controls (76, 77).

Exosomes are membrane-enclosed vesicles released into the extracellular space by all cells and are likely to enter the vascular system. Thus, proteins specifically enriched by cancer-associated exosomes in body fluids may serve as markers for reliable detection of cancer (Fig. 3*C*). To overcome the heterogeneity of common exosomal biomarkers (*e.g.*, CD9, CD63, and CD81) and to identify a universal exosome biomarker, Kugeratski  *et al*. performed super-SILAC-based proteomic characterization of exosomes isolated from 14 human cell lines, including the PCC line PANC-1, PSC, and normal pancreatic cell line HPDE (78). In total, they identified 1212 proteins, from which 22 proteins were universally enriched in exosomes of all cell lines, with syntenin-1 as the most abundant protein. Furthermore, syntenin-1 was validated to be universally enriched in 100 human plasma-derived exosomes, demonstrating the universal utility of syntenin-1 as an affinity target for the purification of plasma exosomes. Another study performed proteomic analysis of exosomes isolated by ultracentrifugation from cancer cells, fibroblasts, and nontumorigenic epithelial cells (79). More than 200 proteins were identified from exosomes isolated from each cell line, and GPC1 has specifically identified in cancer cell-derived exosomes, which was verified in the serum of a large cohort of patients with PDAC (n = 190 and 56 in the discovery and validation cohorts, respectively) and healthy controls (n = 100 and 20) as a potential early diagnostic biomarker with absolute specificity and sensitivity (AUC = 1.0). A recent study profiled the proteome and phosphoproteome of exosomes isolated from the CM of individual tumor epithelial cells and stromal cells and from their co-culture system (80). Around 500 proteins and phosphoproteins were identified in each condition, and some proteins and signaling pathways were exclusively identified under co-culture condition, providing clues of TME remodeling under cancer-stroma interaction. Two proteins (KIF5B and SFRP2) were validated on tissue microarrays as promising biomarkers for early detection and progression evaluation of PDAC.

To identify PDAC-specific surface exosomal markers, Castillo *et al*. performed ultracentrifugation to isolate exosomes from two nonneoplastic human pancreatic epithelial cell lines and 13 human PCC lines, and then exosome surface proteins were biotinylated with a sulfo-NHS-SS-biotin probe for enrichment and MS analysis (81). In total, 7086 proteins were identified, corresponding to 3663 genes, and 6 (EPCAM, CD151, CLDN4, LGALS3BP, HIST2H2BF, and HIST2H2BE) were screened out as PDAC-specific exosome surface proteins, which were used as immunocapture targets to capture cancer-derived exosomes from liquid biopsies. In another study, through comprehensive proteomic analyses of antigen-antibody complexes isolated from plasma and exosomes isolated from PDAC cancer cell lines and plasma, Capello *et al*. demonstrated that the surface membrane of tumor-derived exosomes displayed a large repertoire of tumor-associated antigens, which could attenuate complement-mediated cytotoxicity directed at tumor cells. Two exosome surface proteins (PKM2 and LGALSBP3) specifically enriched in exosomes of plasmas from patients with early-stage PDAC relative to healthy subjects might also serve as potential PDAC biomarkers (82). Apart from discovering diagnostic biomarkers, exosome has also been proven to be an effective therapeutic tool. For example, proteomic analysis revealed that CD47 was specifically enriched on exosomes but not liposomes isolated by ultracentrifugation from human fibroblast cultures, and CD47 positive exosomes enhanced the uptake of exosomes carrying siRNAs targeting oncogenic Kras^G12D^ into tumor tissues, thus enhancing therapeutic efficacy in mouse models of PDAC (83). Mechanistically, CD47 suppressed exosome clearance by circulating macrophages and monocytes, while CD47 and RAS increased the exosome uptake efficiency of PCCs by enhancing macropinocytosis.

Finally, secretome profiling of CM from cancer cell lines combined with ELISA validation in circulation is a useful strategy for the discovery of tumor-specific biomarkers. Makawita *et al*. compared the CM proteome between PCCs and normal pancreatic cell lines to screen PDAC-specific secreted proteins and then narrowed the list by integration with the proteome of PDAC pancreatic juice (84). Finally, by comparing the results with tissue specificity analysis of publicly available databases, they performed ELISA to successfully validate a panel of five proteins (AGR2, OLFM4, PIGR, COL6A1, and SYCN) elevated in plasma samples from 20 patients with PDAC compared with 20 healthy controls. A recent study performed secretome and global proteome comparisons between normal pancreatic ductal cells and PCCs (85). The expression of several proteins (EGFR, GDF15, TGM2, LIF, MX1, STAT1, and SERPINB5) was further validated by PRM. In summary, regardless of the vast dynamic range issue, global and targeted plasma proteomics have identified and validated biomarkers or biomarker panels that could improve the sensitivity and specificity of CA19-9. Moreover, the isolation of exosomes from plasma and secretome profiling of PDAC cell lines could increase the chance of identifying PDAC-specific biomarkers. A brief summary of reported biomarkers may be found in Table 1. Further research is needed to evaluate these biomarkers in large patient populations through multi-institutional collaborations and standardized techniques. In addition, the development of new technologies to overcome the huge dynamic range of the plasma proteome for the discovery of novel PDAC biomarkers remains a daunting task.

**Table 1:** Summary of reported biomarkers covered within this review

Biomarker/Biomarker panel	Proteomic quantification methods	Validation techniques	Sample types	Potential utility	AUC	References
LIF	Dimethyl labeling, LFQ	ELISA, PRM-MS	CM, tissue, plasma	Diagnosis, monitor chemotherapy response	0.848	Shi el al. (43)
A panel of 24 proteins	SIS-MRM-MS	MRM-MS	Tissue	Prognosis, stratify risk subgroup Classification	NA.	Son et al. (57)
CD14, GPC4, ANXA11, and CD44v6	LFQ	Western blot	Organoid, plasma	Diagnosis	NA.	Huang et al. (63)
ANXA1 and ANXA10	LFQ	IHC	Tissue	Diagnosis	0.907 (ANXA1), 0.829 (ANXA10)	Padden et al. (68)
A panel of 14 proteins	SIS-MRM-MS	MRM-MS	Plasma	Diagnosis	0.953–0.977	Kim et al. (74)
ITIH3, APOA1, APOA1, APOL1, and CA19–9	iTRAQ	SIS-MRM-MS	Plasma	Diagnosis	0.99	Liu et al. (75)
TIMP1, LRG1, and CA19–9.	LFQ	ELISA	Plasma	Diagnosis	0.949 (TIMP1), 0.887 (LRG1)	Hanash et al. (76, 77)
GPC1	LFQ	Flow cytometry, ELISA	Exosomes isolated from CM and plasma	Diagnosis	0.781–1.0	Melo et al. (79)
KIF5B and SFRP2	LFQ	IHC	Extracellular vesicles isolated from CM, tissue microarray	Diagnosis	NA.	Jacob et al. (80)
AGR2, OLFM4, PIGR, COL6A1, SYCN, and CA19–9	LFQ	ELISA	CM, pancreatic juice, plasma	Diagnosis	0.93–1.0	Makawita et al. (84)

## Conclusion and Future Prospects

Although MS-based proteomics still faces low sensitivity and throughput compared with transcriptomic analysis based on advanced sequencing technologies, it is widely recognized as an indispensable and powerful tool to discover biomarkers and elucidate molecular mechanisms by quantitatively measuring proteins, PTMs, and their associated protein complexes on a large scale. As described in this review, many achievements have been made by the application of proteomics in functional and clinical research on PDAC, a dreadful disease with limited therapeutic options. Examples include combinatory functional proteome profiling to interpret tumor-stroma interactions and nominate therapeutic targets, spatial proteomics to interpret PDAC tumor heterogeneity, and plasma proteome profiling to identify diagnostic and prognostic biomarkers for PDAC, especially by isolation of exosomes to increase the specificity of biomarker candidates. Further clinical translation of these proteomic discoveries will require a high level of expertise across multiple disciplines, including technology development, medical oncology, pathology, and clinical trials. Anticipated future development and application of innovative proteomic technologies in PDAC includes, but is not limited to, further increase in sensitivity and throughput for analyzing large-scale sample cohorts, high-resolution spatial proteomics analysis of multiple cell types in the PDAC TME, and multidimensional proteomic exploration of PDAC.

Molecular characterization to stratify histopathologically indistinguishable tumors into molecular subtypes is critical to refine therapeutic regimens for precision medicine based on large sample cohorts. However, because of the absence of effective early diagnosis and targeted therapies, it is challenging to access PDAC clinical samples in the early stages of the disease. In addition, to avoid tissue degradation due to the rich expression of proteases, PDAC tissue samples must be properly handled, stored, and processed. To date, only one recent proteogenomic study has performed proteomic profiling of PDAC with a sample cohort larger than 100 patients (35). Therefore, MS-based large-scale profiling of proteins, PTMs, and protein complexes to interrogate defined medical questions of PDAC will be valuable in further research endeavors with clinical cohorts of patients with different treatments, different histopathological stages, and longer well-designed clinical trials. A critical step will be the collection of large sample cohorts with high sample quality to guarantee high-quality data generation and detailed clinical information for fully interpreting proteomic data, such as survival time.

A representative hallmark of PDAC is the typically abundant stroma comprising various cell populations with significant heterogeneity. Thus, another hot research area would be the spatial proteomics of PDAC with higher spatial resolution and deeper proteome coverage. As only nanogram range of proteins could be extracted from LCM sections with enough spatial resolution, it is critical to reduce sample loss and increase sensitivity by using advanced sample preparation and LC-MS technologies. For example, we devised and optimized an integrated proteomics sample preparation technology SISPROT for LCM-based spatial proteome profiling of cancer tissues (86). Moreover, we developed an IHC-based SISPROT workflow to increase the accuracy of spatial proteomic analysis of formaldehyde crosslinked tissue samples (87). In addition, we developed an integrated glycoproteomic approach for spatial glycoproteomic profiling of tissue sections with high sensitivity (88). Furthermore, artificial intelligence (AI)-based automated image processing algorithms for accurate identification of specific cell types, high throughput, and automated sample collection and processing to rapidly analyze large sample cohorts will play an important role in spatial proteomic analysis of PDAC (89). In addition, scRNA-seq and spatial transcriptomics have been used for transcriptomic analysis of PDAC cell type heterogeneity, such as identifying different CAFs subpopulations (90, 91, 92), delineating intratumoral heterogeneity of ductal subtypes (93), mapping tumor and peripheral blood immune landscapes (94) and revealing spatially restricted cell types and subpopulations (95). By integrating these transcriptomic approaches, proteomic elucidation of the cell type heterogeneity at cell type and even single-cell level will provide direct and critical information on molecular functions and identify functionally distinct lineages of cancer and stromal cell types in the PDAC TME.

Finally, we anticipate that a combination of functional and clinical proteomics for the multidimensional exploration of PDAC will be a promising strategy to identify PDAC biomarkers and drug targets. The key rationale is that proteins are the functional biomolecules of the human body, and therefore, there is a higher likelihood that functional proteins will be critical biomarkers and drug targets. It will be desirable to perform clinical proteomic profiling of tumor samples with systematic consideration of protein functionalities in defined signaling networks. Current systematic exploration of proteins and PTMs can only interpret the signaling nodes of various signaling networks. To better understand the protein machinery in defined intercellular signaling pathways, it is highly desirable and challenging to conduct protein complex profiling directly from the same tumor sample. We previously developed a chemical proteomic approach, termed Photo-pTyr-scaffold, which can directly capture and crosslink pTyr signaling complexes for downstream affinity purification by biotin tag and proteomic analysis (96, 97). The Photo-pTyr-scaffold has been successfully applied to explore signaling complexes in the TME of Her2-negative breast cancer and explore new targeted therapies against the receptor tyrosine kinase PDGFRB. We are optimistic that further combination of various protein complex profiling approaches with mature global PTM profiling approaches, such as phosphoproteomics and glycoproteomics, will greatly strengthen clinical functional proteomic studies to better interpret the diverse intercellular signaling network in the PDAC TME and lead to the discovery of more biomarkers and drug targets for conquering pancreatic cancer.

## Supplemental data

This article contains supplemental data.

## Conflict of interest

The authors declare that they have no known competing financial interests or personal relationships that could have appeared to influence the work reported in this paper.
